# Spontaneous Regression of the Pulmonary Metastases in Adenoid Cystic Carcinoma of the Parotid Gland: A Case Report

**DOI:** 10.7759/cureus.30783

**Published:** 2022-10-27

**Authors:** Bidushi Pokhrel, Anusha Chidharla, Prakash Neupane

**Affiliations:** 1 Intensive Care Unit, Hospital for Advanced Medicine and Surgery, Kathmandu, NPL; 2 Division of Hematology and Oncology, University of Kansas Medical Center, Kansas City, USA

**Keywords:** pulmonary metastasis, spontaneous regression of cancer, abscopal effect, parotid tumours, adenoid cystic carcinoma (acc)

## Abstract

Adenoid cystic carcinoma (ACC) is a rare epithelial tumor of the salivary glands with an indolent course and usually bears a long-term survival rate even when metastasized. Spontaneous regression of such a resistant tumor is an even scarce event. We report a case of a patient with ACC of the parotid gland with pulmonary metastases, which spontaneously resolved following resection and post-surgical radiation of the primary tumor. Among the numerous theories proposed to explain such a phenomenon, immunogenic mechanisms and the abscopal effect are the most plausible explanations in this case.

## Introduction

Spontaneous regression of tumors has been occasionally observed in the past; however, it has been rarely known in the case of adenoid cystic carcinoma (ACC) [1]. This phenomenon has mostly been reported in renal cell carcinoma, melanoma, and choriocarcinoma [2]. ACC is a slow-growing malignant tumor of the salivary glands, comprising less than 1% of all head and neck cancers and frequently metastasizing to the lungs and bone. It usually carries an unfavorable prognosis and responds very modestly to treatment, frequently recurring despite repeated and wide excisions [3,4]. However, in the minority of cases, even after the development of distant metastases, patients have been seen to survive for extended periods [5]. For patients with advanced or metastatic ACC who meet the clinical indications for systemic therapy, chemotherapy is offered as an initial treatment. Here, we report a rare and intriguing case of stage IV ACC with pulmonary metastases whose lung nodules spontaneously regressed after the treatment of the primary tumor and had no recurrence.

## Case presentation

In October 2013, a 64-year-old male presented to our clinic complaining of a lump behind his left ear and paresthesia on the left side of his face for two months. On examination, the lump was ill-defined, hard, and non-tender without overlying skin changes. The patient had no signs of facial nerve impingement, sore throat, dysphagia, ear pain or discharge, difficulty breathing, or weight loss. The rest of the physical examination was unremarkable. His past medical history was positive for prostate cancer, which had been resected about two years before his presentation.

**Figure 1:**
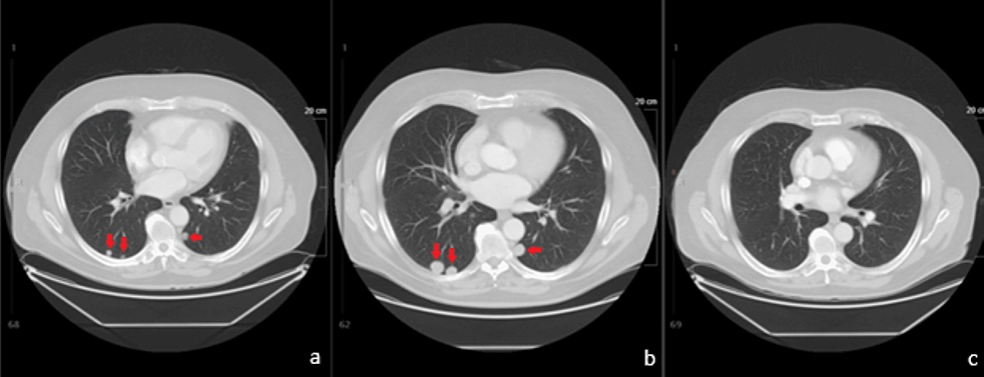
Contrast-enhanced computed tomography scan: (a) before treatment showing multiple pulmonary nodules (red arrows), (b) pulmonary lesions increased at six months following treatment, and (c) the lesions decreased and disappeared completely at 14 months

On his first visit, all the routine blood investigations were ordered, which were within normal limits. The CT scan of the head and neck demonstrated a lobular mass within the superior deep lobe of the left parotid gland measuring 1.9 cm x 2.7 cm x 2.45 cm. Next, a fine needle aspiration (FNA) was performed, with the pathology report favoring pleomorphic adenoma. The patient underwent left total parotidectomy, following which the frozen section was concerning for malignancy, thus necessitating left supraomohyoid selective neck dissection. The final histopathologic evaluation revealed ACC with negative margins and negative lymph nodes with no evidence of residual disease. On further evaluation of the CT chest, multiple pulmonary nodules were seen on both lungs (Figure 1, Panel a), the largest one measuring 1.6 cm in the right lower lobe, with no other distant metastases. The positron emission tomography (PET) scan reported no metabolic activity, consistent with the indolent nature of ACC. On the biopsy of the right lower lobe lung nodule, cytology consistent with metastatic ACC was confirmed.

The patient further underwent parotid and neck irradiation with a total dose of 30 Gy in a hypofractionated course of five fractions on alternate days; however, no radiation to the lung nodules was performed. On subsequent follow-up appointments, the patient did not complain of any symptoms pertaining to the pulmonary metastases. There was residual numbness in the operated left parotid area, but no recurrence of mass or swelling was noted. The patient had some ear discharge, possibly because of radiation necrosis and concurrent external auditory canal infection but mentioned no other active symptoms. On surveillance CT scans of the lungs performed every six months, the nodules notably increased in size for the first six months following the treatment of the primary tumor (Figure 1, Panel b). However, they started regressing in the subsequent scans and completely disappeared 14 months later, without any systemic therapy (Figure 1, Panel c). Currently, nine years following the initial diagnosis, the patient is healthy and in complete remission from the disease.

## Discussion

ACC has been mentioned in the literature as a rare epithelial tumor, having a long natural history, a high tendency for local recurrence (39%), and frequently metastasizing distantly rather than regionally via lymphatics in contrast to most other cancers [6]. Arising commonly in the major and minor salivary glands of the head and neck, it makes up about 6% of all salivary gland tumors [7]. Although mucoepidermoid carcinomas comprised 44% of all malignant parotid neoplasms, ACC was the malignant tumor that is most often encountered in submandibular and minor salivary sites (35%) [6].

ACC was more frequently seen in the female population in the fifth and sixth decades. Pain is the predominant complaint that starts almost before the appearance of a visible mass. In a minority of patients, facial nerve palsy has been reported, likely due to its propensity to cause perineural invasion or even just mechanical pressure [7]. Our patient presented with a noticeable swelling on his parotid gland that progressed over time, but it did not cause any pain or facial palsy. However, there was paresthesia on the involved side of the face, which likely signifies nerve impingement. In addition, he had distant metastasis without lymphatic or regional spread, which illustrates the usual behavior of ACC [3].

Although our patient underwent surgical resection of the primary tumor followed by the tumor bed radiation, no treatment was done for the biopsy-proven lung metastases. This conservative approach was chosen because many studies have shown only a modest response to the treatment of metastases in ACC as the outcomes are not changed with or without treatment [8,9]. Furthermore, the relative ineffectiveness and toxicity profiles of chemotherapeutic agents make it questionable to employ them for a tumor, which is quite indolent in behavior regardless of the outcome. However, surgical removal with wide margins is the preferred modality of treatment of the primary tumor, with adjuvant radiation being usually favored for its ability to promote tumor regression and symptom control [7].

Nevertheless, astonishingly enough, the spontaneous regression of the pulmonary metastases that started to occur six months later in this patient is a phenomenon that has been rarely reported in cases of ACC [1,2,9], and the statement holds true for any other cancer as well. Even then, the most frequently described cancers that have undergone spontaneous regression are renal cell carcinoma, choriocarcinoma, neuroblastoma, and malignant melanoma [2,10].

The frequency of spontaneous regression of cancer occurrence has been predicted to be once in every 80,000-100,000 cases. The postulated theories behind such an event are deprivation of hormonal growth influence in hormone-sensitive cancers, complete surgical removal, unusual sensitivity to inadequate irradiation, fever and/or acute infection, removal of the carcinogenic stimulus, interference with the tumor’s nutrition, and allergic reaction to the tumor cells to name a few [10]. Cole, in his study published in 1981, has majorly attributed immunoglobulins to be the main entities playing a role in cancer regression [11]. The use of vaccines such as bacillus Calmette-Guérin (BCG) has been credited with stimulating similar immunogenic processes, which ultimately potentiate the activity of cytotoxic (CD8+) T cells, natural killer cells, and macrophages [12].

In our case, the patient developed regression of the metastases following treatment of the primary tumor. The plausible mechanism here could be immune-related as well. This could further be explained by an entity called the "abscopal effect," bystander effect, or out-of-field tumor response, wherein tumor regression occurs in a site distant from the primary site of radiotherapy. Local radiotherapy has been known to trigger immunogenic cell death, thereby leading to certain host immune responses. When radiation hits the tumor cells, there is a release of proteins called damage-associated molecular patterns (DAMPs), which induce the engulfment of antigens by the antigen-presenting cells, consequently resulting in the improved activity of cytotoxic immune cells [13].

In most cases reported, radiation and immunotherapy combined have been observed to produce such an effect, unlike in our patient who did not receive any form of immunotherapy. Previously, the abscopal effect has been demonstrated in the case of ACC in a few case reports [14]. The Danish and British Columbia trials demonstrated that postoperative radiotherapy for high-risk breast cancer patients reduced the risk of loco-regional as well as distant recurrence considerably [15,16]. Even in these trials, control of the distant metastases could be attributed to the abscopal effect exerted by the radiation on the tumor bed containing microscopic or residual disease. Thus, in our patient, we can hypothesize that the abscopal effect following radiation to his primary tumor bed triggered a heightened immune response for the clearance of metastases.

## Conclusions

We have reported a very rare case of spontaneous regression and durable complete response of metastatic sites in ACC, which is a remarkable event from an oncological perspective. No mechanism can sufficiently explain this event; however, the most plausible is the immune-mediated and abscopal effects of radiation. Further exploration of these rare events and mechanisms could help in discovering effective therapies to target stubborn cancers like ACC in the future.
